# Green Lungs and Green Liberty: The Modern City Park and Public Health in an Urban Metabolic Landscape

**DOI:** 10.1093/shm/hkac055

**Published:** 2022-09-24

**Authors:** Karen R Jones

**Affiliations:** School of History, Rutherford College, University of Kent, Canterbury CT2 7NX, UK

**Keywords:** park, urban, environment, health, city

## Abstract

This paper explores processes of urban park creation from the mid-1800s to show how ‘green lungs’ and ‘green liberty’ shaped the health geography of the modern city. Tracking this story across a transatlantic canvas (using examples from London, Paris, New York and Montreal), it looks at how ideas around fresh air, exercise and greenery sat within municipal designs for a functional metabolic landscape, what I call somatic urbanism. Plotting the historical contours of the park as a landscape of health has two main uses. First, it usefully connects the worlds of medicine and environment to show how debates about industrialism, modernity, sanitation and social reform found common ground. Second, in a contemporary world where ventilation issues have been highlighted by the coronavirus pandemic and municipal authorities grapple with anthropogenic challenges, it argues that historical studies of health and environment assume a vital importance in shaping the future of sustainable cities.

In his 2005 article ‘In Search of Health: Landscape and Disease in American Environmental History’, historian Gregg Mitman argued for a new attentiveness to the worlds of health and medicine in excavating human–nature interactions in the past. Connected discourses of ecosystem and human well-being, he noted, were to be found across the historical canon, from the classical humours of Hippocrates to the musings of twentieth-century American conservationist Aldo Leopold, whose ‘land pathology’ set out in *A Sand County Almanac* entwined medical and conservationist thinking to create a holistic vision of functioning systems. Strange then, Mitman pondered, that health has ‘not been a subject more central to environmental history’. He attributed this to various oppositional categories that served to separate the realms of western medicine and environmental science: human/nature; local/global; urban/rural. I would add to this a twentieth-century medical praxis that focussed on interior space as a locus of professional expertise (laboratory, hospital) and thereby created a false dichotomy between the health geographies of the ‘great outdoors’ and the ‘great indoors’.^1^

Since Mitman’s call, various scholars have deconstructed the boundaries separating human and environmental health to create a rich tapestry of work that apprehends the modern city as a complicated organic space shaped by ideas of pollution control, multi-species choreography, sanitation hydrology and disease epidemiology. Here, I navigate a similar course with reference to the park, pointing especially to the synchronicities of human and ecosystem health that marked the emergence and evolution of this ubiquitous slice of landscape architecture. Illuminating both the aspirations and contestations of the urban world, green space, in the words of border studies specialist Araceli Masterson-Algar, offers a valuable ‘text into the city’. For historian Peter Clark, it represents ‘a fundamental concept for understanding modern and contemporary urban society…the ecological development of cities…societal relations, urban governance and planning’. Of particular significance for this study are the entangled discourses of health, modernity and industrialism, specifically those that conjured the park as an essential spatial medication.^2^

In the phase of urban park-making on a grand scale which spanned the mid-nineteenth until the early twentieth century (labelled by urban theorist Galen Cranz as the era of the ‘pleasure ground’), a diverse collection of planners, philanthropists, civic leaders, medical experts and social reformers looked to the park as an important device for the promotion of public health and community betterment. The notion of parks as ‘lungs for the city’ is well known and their provenance as sites designed with health in mind widely cited in the literature on landscape architecture. What this article does, however, is to dig deeper into this planting for health rationale with a more consciously environmental lens and somatic frame of reference. A focus on the body, as feminist theorist Elizabeth Grosz posits, is useful for its ability to travel across material and metaphorical planes and to ‘problematise the opposition between the inside and the outside’. Grosz spoke principally of traversing interior and exterior realms in terms of human-city navigations, but her intervention is also useful in terms of transgressing the boundaries of medico-environmental place-making. Turning specifically to parks, I argue that they served as both preventative (sanitary) and curative (medical) devices, ones through which a new cadre of urban interests sought to tackle the modern city as a holistic environmental and social organism. Their solution to the challenges posed by a dynamic and rapidly growing built environment was circulation—the free movement of atmospheric, hydrological and organic elements—and focussed on two specific purposes for the park. The first of these concerned its ability to breathe life into the urban body as a ‘green lung’—a medical metaphor which served both as abstraction, a useful civic strap-line, and accurately expressed practical functions of ecosystem and respiratory services. The second drew on long-standing associations between land, rights and personal expression and incorporated the communitarian, utilitarian and philanthropic impulses of the hour to frame new urban infrastructures of greening as worthy repositories of ‘green liberty’. Rescued from elite genealogical roots and its gates thrown open to all-comers, the park re-positioned itself as a progressive experiment in democratic public amenity.^3^

The powerful allure of the city park as a ‘green lung’ and locus of ‘green liberty’ saw it take root as a locus of urban well-being across a global topography. An active space where material and metaphorical ideas about nature, society and well-being could be articulated, recalibrated and transplanted, the park became a kind of all-purpose spatial prescription, a place where metropolitan authorities wrestled with both the opportunities and the challenges created by the transformative forces of urbanisation, industrial capitalism and mass democracy. It was, in short, an experimental place where the paradox of modernity—the city as a place of aspiration and anxiety, what Richard Dennis calls ‘overcrowding and frenetic energy’—could be resolved. Approaching the park as a modern urban artefact might strike as an oxymoronic contention given the template of pastoral lawns and winding pathways that crafted them (often regardless of location) as bucolic escapes to the British countryside. These design contours represented nothing more than naturalistic conceit. Instead, park space was meant to sustain the city by a powerful injection of green tonic: to heal its ‘growing pains’ and to guarantee a glittering urban future through operational ornamentation. Writing in *The Granite Garden* (1984), Ann Whiston Sprinn duly hailed nineteenth-century park planners for their visionary approach to urban ‘future-proofing’ in addressing issues of climate, pollution and flooding, while landscape historian Catherine Ward Thompson saw their work as important ‘prototypes’ for urban design today in terms of its blended approach to socio-environmental well-being. At the same time, however, the park presented an imperfect remedy for the urban condition. A trip to the ‘pleasure ground’ may have promised fresh air and perambulation, but, as Peter Thorsheim notes, it was also there to discipline working class minds and bodies. Beneath the lofty ideals of unbounded and restorative greenery, a knotty landscape of power relationships, paternalism and social inequalities shaped the nature and functionality of urban green space.^4^

What follows is a survey of the health genealogy of the city park that foregrounds the importance of (what I call here) somatic urbanism, a holistic vision of living cities that incorporated medical, humanist and planning perspectives on the eco-social dialectics of space. It begins with a conceptual skeleton (therapeutic landscapes, embodiment and urban metabolism) which serves as an interrogative mechanism for the analysis of urban infrastructures of well-being, before moving on to dissect the anatomical fixings of the park and its emerging role in sustaining the city body. Drawing on examples from four cities—London, Paris, New York and Montreal—the next two sections track the ways in which the dual branches of ‘green lungs’ and ‘green liberty’ created a powerful schema of planting for health that took in both old and new park variants across a transatlantic geography. The cities discussed here were not the *only* places where parks were created in this period, but they were prime sites for the exercise of park-making and ones that consciously aligned the creation of green space with citizen health, urban planning and the workings of what Martin Melosi calls ‘the sanitary city’. Having looked at ideology, implementation and international transmission, focus turns to everyday use of park space and the ways in which, as Catherine Ward Thompson puts it, ‘the democratic process worked out on the ground’. Significant here were the spatial, vernacular and socio-economic constraints that shaped the citizen experience with public amenity. Rights to wander democratic pathways did not mean wandering unrestrainedly. The study ends by revisiting this story through a twenty-first century lens to point to an ongoing conversation about urbanism, sustainability and health. Playing out in parks today are challenging issues around the privatisation of leisure; social exclusion and health inequity; anthropogenic threats to ecosystem resilience; and unstable political economies of management—each of which are usefully contextualised by understanding the historical role of the park as a critical health geography.^5^

## Conceptual Frameworks: Therapeutic Landscapes, Embodiment and Urban Metabolism

To better understand the health geography of the modern city (and the place of the park in it) we need to look at how ideas around eco-cultural wellness (broadly conceived) informed the rhetorical turns, grand visions and design schemes of landscape designers, municipal officials and urban planners. Three theoretical concepts present helpful guides to action in mapping the somatic choreography between an emerging park movement and the booming architecture of the modern industrial city—from geography, the notion of therapeutic landscapes; ideas around embodiment, as articulated by scholars of medical humanities; and, from science and technology theorists, conjurings of the city as an urban metabolism. Each are worth detailing in brief.

First, the terminology of therapeutic landscape as developed by health geographer Wilbert Gesler. Gesler developed this idea as a ‘geographical metaphor for aiding in the understanding of how the healing process works itself out in places’, combining medical and cultural geography to explore the ways in which physical, social and perception aspects conspire to create spaces imbued with a sense of healing. However, where Gesler’s focus was on exploring spatial habitats for the sick (from hot springs to hospital consultation rooms), the park represents a multi-functional medical environment—one that applied the healing properties of pastoral landscape to revive the city at large as well as individual citizens and served as both preventative and curative. More recent interventions from health geographers have refined Gesler’s framework to deconstruct the ways in which place, body, society and individual formulate different concepts and capabilities around wellness and spatial engagement. This new attentiveness to experiential encounter and on taskscapes and mobilities offers a useful methodological springboard to explore the everyday interface between people and parks in the past. How might notions of movement—in terms of flow, activity and barriers—deepen our understanding of the civic healing experience in a vernacular landscape?^6^

Complementing this perspective is a focus on the body which draws inspiration from the medical humanities. Research in garden history and the history of medicine has highlighted the role of garden areas in hospitals, recuperative spaces and asylums, and this article builds on these foundations to articulate a sense of the city at large as a holistic medical body, one which was being dissected by a diverse set of constituencies interested in public health, ventilation and the place of green space in a wider health-landscape nexus. Although work on the sanitary organisation of the modern city is extensive, dedicated green space has been only loosely connected to medically framed perspectives that tend to look at disease modelling, engineering systems and epidemic anxieties. Equally important is the way in which academic scholarship on embodiment sheds light on the relational dynamics of park landscapes to pose valuable questions about user groups and the nature of their feelings about urban green space. What was it like to spend time in newly established parks? How were citizens transformed by their engagement with its curative paths and shady arbours? A brief glance at the historical record suggests that sensory cues consistently informed discourses on the park: taking a breath of pure air, observing the undulations of pleasing scenery, hearing not the hubbub of the city or smelling its olfactory outbursts but instead enjoying silence and the scent of flowers or trees. Movement, especially walking, played into the equation in decisive ways as an activity invested with a multitude of practical and imaginative benefits. Approaching the park, then, as a whole body experience, informed by various sensory aspects and cathartic exercises, adds a fresh dimension to deconstructing the health pathology of the city park.^7^

Joining the methodological blend is an intervention from engineering and science and technology studies that injects an important spatial element into the study of park-making in the modern city and provides a useful theoretical base for exploring its function within an organic system of resource transfers, socio-ecological interactions and energetic movements. As Richard Sennett explains in *Flesh and Stone*, early modern anatomist William Harvey’s research on blood and physiology found natural transplant in the conceptualising of a new urban world, the notion of ‘flowing arteries and veins’ a helpful model to work with in designing thoroughfares and infrastructures for growing urban conurbations. Pamela Gilbert argues that urban planners from the mid-nineteenth century eagerly conjured with ideas of the city as a dynamic ‘organism with its own rhythms and cycles’, while Richard Dennis describes the networking of the industrial metropole as an intrinsic part of what make it modern. This emerging sense of the bodily contours of the city, a somatic urbanism, presented a critical meeting point where the metaphorical and the material, the quantitative and the ideological, could mix. Particularly helpful in excavating the terms of this imaginative and physical transaction is the concept of urban metabolism. Developed as a model to describe the flows and interactions between human and natural systems, the idea borrows from sociology, biology and urban theory to describe the city as a connected entity in which the dynamics of power, production and growth played out. Conceived by engineer Abel Worman in the 1960s to describe movements of water flow, waste and social inequities in American cities, the term is most commonly associated with civil engineer and historian of technology Sabine Barles and her work on energy systems, social relations and environmental pollution. Thinking specifically about park-making campaigns, meanwhile, urban metabolism seems especially valuable in helping to contextualise the anatomical designs of planting for health and the value placed on it to perambulate fresh air, water and people within a vibrant city body.^8^

## The Anatomy of a Park: Modernity, Circulation and the Lungs of the City

In 1750 London’s population was 700,000. In 1825, it reached 1,350,000. By 1901, the city boasted more than 4,500,000 residents. Paris grew from 546,000 in 1801 to 2,700,000 a century later. Advances in industrial technology, economic growth and mercantile power transformed these nineteenth-century capitals into dynamic and expanding world cities. Political status, financial power and cultural prestige emanated from their vertically and horizontally spreading environments of factories, civic buildings, commercial and residential districts, creating in the process an energetic and hungry urban metabolism. Dr Neil Arnott in *Elements of Physics* (1833) described the relationship of water flowing through pipes from the Thames as analogous to blood fuelling the human body, while Victor Hugo saw the passages of subterranean Paris as a ‘dark network’ of arteries, an ‘intestine’ of the city in need of rational organisation. This biotic referencing was pertinent. Across various philosophical, professional and political constituencies in the modern city, bodies of various design (civic, medical and social) were being chewed over in the context of an expanding urban industrial system, and, especially, in conjunction with debates about sanitary reform and municipal responsibilities. As social historian Patrick Joyce notes, ‘The sanitary economy of the town was like that of the body. Both were characterised by a dynamic equilibrium between living organisms and their physical environments’. Significant in this equation was the fact that billowing factory chimneys and galloping urbanisation began to emerge as spatial markers of the devastating consequences of unbridled industrialism: overcrowding, social alienation, poverty, disease and pollution. This was the modernity paradox—the urban environment as monster and moderniser, chaotic and energetic—which underscored calls to heal the elemental afflictions of the city as it grappled with new infrastructures of home and work, forms of production and cultural relations. Architect Henry Roberts, writing in *Home Reform: Or What the Working Classes May Do to Improve their Dwellings* (1852), saw urbanism as an all-encompassing ‘physical and moral malaria’ which was ‘wafted abroad by the winds of heaven to pollute and to poison whatever falls within their reach’, while Charles Dickens looked to bodily reference as an evocative way to depict London on its last legs in *Our Mutual Friend* (1865), conjuring the city as an animate entity choking up ‘a gigantic catarrh’. Speaking of Paris in similar tone, social reformer Victor Considerant complained of an ‘immense workshop of putrefaction, where misery, pestilence and sickness work in concert, where sunlight and air rarely penetrate’.^9^

It was in this context of a sick city suffering from a litany of public health problems that municipal reformers scrutinised the ecological fabric of urban life, looking particularly at the free movement of fresh air, clean water and sunlight as essential vectors of a healthy city geography. These formative enquiries into somatic urbanism found a remedy in circulation, an all-purpose solution that seemed to speak to various motors of prosperity in modern city life, from chemistry to political economy, engineering to philosophy. In 1829, botanist and garden designer, John Claudius Loudon published a radical redesign of London’s built environment based on the need to rationalise city expansion and facilitate the expeditious movement of organic materials. Entitled ‘Hints for Breathing Places’, it presented a concentric restructuring of metropolitan roads based on what Loudon called ‘ready and economical intercommunication’, social inclusion and access to recreational space. His vision imagined a ‘circle of turf’ at the centre of the capital, with a half-mile wide green belt further out, based on the idea ‘there would never be an inhabitant who would be farther than half a mile from an open airy situation, in which he was free to walk, to ride, and in which he could find every mode of amusement, recreation, entertainment and instruction’. Issues of communicative disease were particularly instrumental in encouraging a closer look at spatial dynamics. The early 1830s saw the first outbreaks of cholera in London and Paris, at once prompting widespread concerns about the transmission of epidemics across a dense and overcrowded cityscape and inviting a sanitary reading of urban space. Various letters to *The Lancet* in this period emphasised the causal link between healthy bodies, poverty and effluents, while, on taking office immediately after the cholera episode that killed 19,000 Parisians, Prefect Rambuteau positioned his ‘first duty’ as providing ‘water, air and shade’ to metropolitan citizens via tree plantings designed to ‘restore the eyes and purify the air’. Subsequent outbreaks in the late 1840s and early 1850s cemented a sanitary reading of the built environment and the sense of an urban metabolism under attack. Accordingly, government bodies, medical professionals and social reformers argued, the somatic health of the modern city demanded an encompassing set of environmental, technical and social prescriptions. In London, this was demonstrated by the expansive portfolio of the Metropolitan Board of Works (1855–89), which created 80 new sewers, pulled down more than 7,000 high-density tenements and constructed the Thames Embankment with flow and circulation in mind. In Paris, likewise, Georges-Eugene Haussmann’s grand redesign of the city (1853–70) looked to address issues of congestion and disease by the creation of wide thoroughfares that allowed for ventilation and movement. Viewed through a holistic lens of environmental and social hygiene, concerns over population growth, social hygiene, mortality and infection worked in concert to demand a wholesale remaking of urban topographies. In this expansive ‘medico-moral discourse’, as Clare Hickman describes it, the role of green space became a matter of particular consideration. Prominent British public health campaigner, Edwin Chadwick thus included a section on the social benefits of Derby Arboretum (1840) in his ground-breaking commentary, *The Sanitary Conditions of the Labouring Population of Great Britain* (1842), pertinently titled ‘Effects of Public Walks and Gardens on the Health and Morals of the Lower Classes’. Meanwhile, for Jean-Charles Adolphe Alphand, landscape architect under the employ of Napoleon III, squares, parks and street trees represented essential inclusions in the terrestrial remodelling of Paris in order that ‘air can circulate freely’ in the ‘cause of sanitary conditions’. As the modern city expanded at pace, long-held associations of bucolic nature as a healthful refuge that stretched back to the classical world readily assimilated into a political and professional dialogue that looked to municipal planning, science and sanitation as the building blocks of a successful urban future.^10^

The most striking example of this blended environmental–medical conversation was to be found in articulations of the park as a functioning respiratory system. Popularly attributed to William Pitt and first mentioned in parliamentary debate in 1808 about urban development in the West End, Hyde Park was hailed as the ‘lungs of London’. With the capital beginning to suffer from the beginnings of urban sprawl and from industrial pollution, park defenders saw the purpose of green space as twofold. First, the open spaces and trees in such preserves served to ventilate the city, circulating the air and thereby preventing it from becoming stale and stagnant. Advocate John Windham in parliamentary debate celebrated what he called ‘the power of vegetation’ to cleanse surrounding air. Equally important was the function of green space in providing ‘health and recreation’—the movement of people through a salubrious environment by walking or riding in healthful surroundings in order to ingest its medicinal benefits. As Loudon put it, London’s parks and squares remained ‘of greatest consequence to the health of its inhabitants’ as anatomical and atmospheric purifiers. Underscoring this attention to circulation was the idea of ‘bad’ or foetid air, the miasma theory (which had held sway since the time of the ancient Greeks) stipulating that rotting organic matter from human or animal sources created noxious vapours from which diseases were carried and communicated. The cholera epidemic which scourged London in the 1850s was famously attributed to bad air accumulating near the Thames by medical professionals including Dr William Farr, assistant commissioner for the 1851 Census. Although John Snow’s study of the Broad Street pump made an epidemiological breakthrough in identifying the outbreak as water-borne, the minutiae of his theory was overlooked by the medical community until Louis Pasteur and Joseph Lister’s work on germ theory in the 1860s and 1870s. In the meantime, the focus of sanitarian concern remained with the dangers of miasma and the removal of dank environments where toxic or ‘corrupted’ air congregated. Land reclamation of marsh, swamp and ditches and the creation of open ‘airing grounds’ thus combined landscaping acumen and medical diagnosis to tackle the most persistent and infectious threats facing the modern city. Robert Angus Smith, who ran the British Alkali Inspectorate from its inauguration in 1864, hailed the medical benefits of parks as vital lungs that enhanced both ventilation and air quality: they ‘allow the wind to blow around us during the day, and…supply [fresh air] during the night’. Also worth noting was the view held by Chadwick and a broader public health constituency that ‘smell’ equated to disease, a powerful demonstration not only of the potency of the zymotic concept of illness transmission but also an evocative example of the sensory dimensions by which people judged healthy and unhealthy spaces.^11^

## Citizens and Civic Health: Land, Liberty and the Liveable City

The incorporation of city parks into the infrastructure of a modern city typically followed two paths. The first saw a remodelling of older green sites (often royal hunting preserves) into places for all. The second invited the creation of new spaces that were consciously crafted as ‘People’s Parks’. In both instances, the allocation of green space to cater for the needs of a city population mapped out the particularly democratic fixings of somatic urbanism. Joining the medical argument for planting for health, then, was an important corollary: one that cogitated on the rights of urbanites to claim unencumbered access to clean air, water and public space. As such, this new egalitarian infrastructure powered by municipal, philanthropic and sanitary design spoke not only the language of hygienism but also of classical understandings of individual liberty and its relationship to land, civic identity and community virtue. On a practical level, meanwhile, the paradox of modernity could only be remedied if city parks were able to impart their healing powers to the citizen body at large. Put simply, ‘green liberty’ was necessary for the ‘green lungs’ to function to capacity.^12^

In London, the process of democratising the park saw the relaxing of entry requirements to a slew of royal preserves which had long served as enclaves of *rus in urbe* for wealthy socialites, namely Greenwich (1433), St James’s (1532), Hyde (1536) and Richmond (1637). The Regent’s Park, founded in 1811 as part of an elite housing development, opened doors to commoners for 2 days a week in 1835. Lauded as necessary investments in public landownership, green space campaigner Octavia Hill spoke of how parks should be a ‘common inheritance from generation to generation’, while J. F. Murray saw circulations of air and citizens as guarantors of urban democratic vigour in celebrating ‘the lungs of London’ as ‘great vehicles of exercise, fresh air, health and life to the myriads that congregate in the great metropolis’.^13^

Victoria Park, in the East End of London, represented an early experiment in creating a ‘People’s Park’ from scratch. Evident here was a sense of the merging of socio-environmental concerns under the banner of planting for health. Indeed, when the Government’s 1833 Select Committee on Public Walks published its findings, it recommended five parks for London based on a need for ‘Public Walks or Open Spaces, fitted to afford means of exercise or amusement to the middle or humbler classes’. An illustration of the connections between health regimes and the movement of working-class bodies, as well as the striations of green liberty, civic health, leisure and social improvement, the Committee called for the placement of street trees and seating along all new roads to make for a pleasant walk for ordinary folk. At present, it complained, the capital struck as an unhealthy geography bereft of greensward and marked by dusty and dangerous transit routes: ‘[l]eaving the Regent’s Park towards the East…for several miles along the Northern edge of the Metropolis, all the way to the River at Limehouse, there is not a single place reserved as a Park or Public Walk, planted and laid out for the accommodation of the People; yet there is no part of London where such Improvements are more imperatively called for’. As well as healthy exercise, disease prevention via the circulation of atmospheric elements a represented a critical driver. Open ground, advocates argued, ensured the movement of fresh air and acted as a buffer to stop epidemics raging across residential districts; allowed for individuals to undertake healthy mental and physical activity; and reclaimed a slice of land which had been used by so-called unsavoury elements (criminal gangs and political agitators). Civil servant William Farr argued the case for a park in the East End on the basis of public health, noting that it, ‘would probably diminish the annual deaths by several thousands…and add several years to the lives of the entire population. The poorer classes would be benefitted by these measures… But all classes of the community are directly interested’. An 1840 petition signed by 30,000 and delivered to Queen Victoria made similar pleas on the grounds of environmental and social uplift: ‘Poor People, closely crowded in confined districts, have no open spaces in the vicinity of their humble dwellings for air, exercise or healthful recreation; circumstances which produce the most painful effects on their physical and moral condition’. Once more, the miasma theory was raised as an important medical justification, with the lack of effective ventilation seen as encouraging not only disease but also a ‘moral pestilence, which is partly produced, and greatly aggravated by the want of open spaces. Unable to breathe the pure air of Heaven with their families, multitudes are driven into habits of intemperance, bringing in their train demoralisation, disease and death’. The combination of fresh air and green expanse represented a multi-purpose remedy and one which municipal authorities heartily endorsed. In 1841, Minister of the Office of Commissioner of Woods and Forests signed off on the creation of Victoria Park under the remit of improving the general health of London. Park gates opened in 1845.^14^

A similar rationale was evident in the creation of Battersea Park, on the banks of the Thames, where arguments for the establishment of a green enclave centred on the cleaning up of an area of marshy commons known for its stagnant water and its raucous taverns and gambling subcultures. Original plans looked to transform 320 acres by levelling, drainage, the creation of carriage rides and walks, an ornamental lake, rockwork, shrub and tree planting. Drawn up by landscape architect James Pennethorne (who also designed Victoria Park), the masterplan envisaged an attractive river frontage and landing station purposely connected to the new engineered landscape of the Thames Embankment, with adjoining areas assigned for residential housing (the sale of these grand properties, it was hoped, would pay for the park). It took years to raise funds and secure land rights over the patchwork of titles in the area, but when Battersea finally opened in 1858, the horticultural remodelling above ground and sewers below communicated a grand and synchronised vision of civic improvement, sanitation technology and democratic resort.

Park-making in Paris presented a symmetrical endeavour of planting for citizen heath, one that illuminated the trans-manche circulations of aesthetic and sanitary ideas, the distinctions of national as well as metropolitan culture and what Dennis calls ‘the active role of space’ in city life. As in London, previously exclusive spaces threw their gates open to the public. The Tuileries—a place of high society promenading in the time of Louis XV—became a National Garden after the Revolution where the incarcerated Bourbons walked in the morning and the public wandered in the afternoon. Drawing clear lines between public space and the aspirations of *liberté*, *égalité*, *fraternité*, Abbot Gregorie in his *Essai Historique et Patriotique sur les Arbes de la Liberte* (1793) aligned rights to the city with open space and citizen assembly, while in *Promenades, Parcs Jardins Paysagers* (1904), philosopher Ernest Guinier (though complaining about the health irritants of sycamore burrs for those with chest ailments) reflected on the power of greening in a democratic, somatic city, a place where ‘the body resumes its rights in this urban nature’.^15^

While the valorisation of street trees (notably the Justice Tree, adopted as national symbol in 1792) highlighted strong connections between living nature, political freedom and democratic voice in republican France, it was with the Second Empire that park-making received greatest scrutiny. Under the aegis of civic engineering, paternal metropolitanism and public health, stress was placed on circulation, citizenship and sanitation. Accordingly, Haussmann’s designs for Paris envisaged a utopian future along geometric lines, radiating central authority in boulevards that followed in the schematic footsteps of Louis XIV’s Versailles and saw straight lines and rational planning as the best way to distribute materials and matter around a modern city. Spatial features played a critical role in this networked metabolic landscape, trees separating flows of pedestrians from street traffic, squares providing neighbourhood hubs of ventilation and shade, and large parklands—created as organic anchors at the compass points of the city—whose recuperative contours drew not on French formalism but planned pastoral aesthetics in the English style. Vividly depicted in Alphand’s planning blueprints *Les Promenades de Paris* (1867–73), Parc des Buttes Chaumont, situated to the north–east and constructed between 1864 and 1867, illuminated the relationship of the park to urban industrialism and the paradox of modernity: an abandoned gypsum quarry remodelled into a picturesque garden of rustic delight that circulated pedestrians and carriages in separate systems and served as the grand backdrop for the modernist energies of the *Exposition Universelle d’Art et d’Industrie.* The Bois de Boulogne, an ex-royal hunting park in the west, equally showed the park as malleable terrain: its geometric avenues meandered by Alphand into circuitous pathways, lakes and tree berms, which, when completed in 1858, drew direct inspiration from Hyde Park, where Napoleon III had strolled in exile. According to Baron Ernouf in *L’Art des Jardins* (1868), Parisians had been forced to leave the city ‘to breathe air and enjoy the sunshine’, but now the urban world had a ‘restful atmosphere’, with public parks ‘spread more or less evenly through all quarters of the town’.^16^

## Somatic Translations: Park-making and Transatlantic City Bodies

The concept of the park as a vital respiratory device and repository of citizen liberty proved eminently mappable onto an expansive geography, the imaginative contours of anatomical well-being and urban prescription easily transcending national borders and site-specific elements to become part of a global movement of park-making. The use of somatic reference, of course, was easily understood, biological nomenclature being a well-utilised device for explaining human engagement with the world (clock ‘faces’, ‘mouths’ of rivers and the like). Moreover, the idea of urban green space being gifted with the label as ‘lungs’ of the city and mantled with democratic portent was eminently applicable to any conurbation grappling with modern industrialism and its socio-environmental consequences. A case in point was New York, where demographic and economic growth saw the mushrooming of a built landscape that grew from 96,000 citizens in 1810 to 4,766,000 a century later. Concerned about the impacts of urban expansion on the health of residents, the *New York Mirror* in 1842 drew on anatomical allusions to explain how parks and trees served to ‘purify and regenerate the atmosphere, in the same way as the lungs give it to blood, changing in venous blue to an arterial scarlet’, while in 1853, the *New York Daily Times* clamoured for a health-giving parkscape for the residents of Manhattan because ‘a million people will soon be crowded on this island, and if these lungs of the city are not furnished…this will become a sickly place’.^17^

The transatlantic migration of a medicalised park vernacular thus seemed both intuitive and informed, a long tradition of biological allusion given fresh urgency by a new urban pathology. According to North America’s foremost landscape architect, Frederick Law Olmsted, the transnational drive for greening the city sprang from ‘a common spontaneous movement of that sort which we conveniently refer to the “Genius of Civilisation”’. Olmsted’s assessment of the ‘justifying value’ of parks (1880) framed the addition of green space as a naturally developing component of a modern city. At the same time, it is worth mentioning the cross-fertilisation of ideas based on the ecumenical allure of planting for health. According to Sprinn, the germination of parks across old and new city bodies was a congruent process based on the universal appeal of the ‘trappings of nature’ as spatial prescription. Facilitating this transmission was a communicative group of social reformers, humanist thinkers, medical professionals and landscape architects whose conversations on horticulture and human betterment spanned lines of professional expertise and national borders. Rousseau was a keen advocate of British pastoral aesthetics for what he saw as their alignment to natural rights, while William Robinson, famous for his wild gardening style, effused on the Parisian parks as truly democratic plantings. The hygienist community was equally conversational, with debates around epidemiology (especially of cholera) looming large in an expansive public health dialogue around sanitation and disease. Accordingly, when the urban ailments of the New World were compared to the old, the park emerged as a popular salve for the restitution of somatic function. *Scientific American* urged the USA to follow the example of European cities, where ‘there are large public parks which form huge lungs for the pent-up streets’, while Caroline Kirkland returned to New York from a visit to London in the 1840s inspired by ‘oases in the wilderness of streets’ that provided ‘recreation for the weary, the sad, the invalid, the playful’. Equally, when Olmsted toured the UK in the early 1850s, the experience of which was recorded in *Walks and Talks of an American Farmer in England* (1852), his reflections on park variants communicated a sense of social and environmental worlds entwined and of a keen analysis of landscape virtue. While the old deer park at Eaton Hall impressed for its graceful scenery, the power politics embedded in this (and other) exclusive landscapes of wealth did not. In contrast, Birkenhead Park (1843), designed by Joseph Paxton for city residents as ‘entirely, unreservedly, and for ever the people’s own’ prompted effusive praise, as did the model town nearby which impressed for its sanitary facilities. Finishing his travels in London, Olmsted concluded the healthful enclaves of the royal parks to be entirely the best thing about the capital.^18^

Turning to planting for health in a North American theatre, the creation of New York’s most famous green landmark—Central Park—came from a campaign led by city literati, journalists and social critics (notably garden designer Andrew Jackson Downing and editor of the *New York Evening Post* William Cullen Bryant). Having secured most of the land needed for the project, the Central Park Commission launched a design competition in 1857 to solicit visions for the space and drum up popular interest. Thirty-five submissions were received, from which the judges picked a blueprint authored by British architect Calvert Vaux and Frederick Law Olmsted. Under their ‘Greensward Plan’, Central Park was remade as a pastoral refuge where city residents might escape the noise and bustle of city streets to take respite in bucolic scenery. Health concerns were at the heart of Olmsted’s ethos, expressed in this design philosophy and in his extensive landscape portfolio thereafter. Using the nomenclature of bodies, breathing and flow, he consistently located the vital function of parks as ‘ventilating places’ and ‘airing grounds’. Thinking more specifically of the cleansing role of green space, he explained how ‘modern science has beyond all question determined many of the causes of the special evils by which men are afflicted in towns’, a leading example being the poor quantity and quality of air moving through the lungs of an urban citizenry. Parks were thus critical as a spatial restorative, in Olmsted’s words, for giving ‘the lungs a bath of pure sunny air’ and ‘giving the mind a suggestion of rest from the devouring eagerness and intellectual strife of town life’. In this health-landscape, configuration biotic elements performed an essential cleansing role: ‘Air is disinfected by sunlight and foliage. Foliage also acts mechanically to purify the air by screening it. Opportunity and inducement to escape at frequent intervals from the confined and vitiated air of the commercial quarter, and to supply the lungs with air screened and purified by trees and recently acted upon by sunlight…if these could be supplied economically, our problem [i.e. the urban condition] would be solved’. As well as his European travels, Olmsted’s holistic socio-environmental vision of landscape drew on his formative years in rural Connecticut, experiences as a magazine editor living in an incendiary New York City in the 1850s and his career experience as General Secretary of the US Sanitary Commission in the Civil War (1861–3), an appointment which furnished him, as Thomas Fisher notes, with a keen sense of the connections between healthy human bodies and healthy environments.^19^

Realising a vision of medical and environmental uplift in central Manhattan required significant logistics. As Olmsted noted of the site, ‘It would have been difficult to find another body of land…upon the Island [with]…less of the desirable characteristics of a Park’. Five million cubic feet of soil and rubble was removed, the terrain drained and levelled, and a boundary of trees planted around the entire site to create a sense of rural escapism. Modelled on an English landscape aesthetic that emphasised the pastoral and the picturesque, the emerging park featured winding paths, wooded nooks and a large lake designed to create spaciousness and tranquillity. Berms and banks removed all sight of roads (perhaps inspired by the underpass in London’s Regent’s Park, with which Olmsted was particularly taken) and also soaked up the sounds of the city. Carefully designed pedestrian, equestrian and carriage routes ensured the seamless circulation of visitor bodies within park confines. This 700-acre sylvan fantasy in the middle of bustling Manhattan thereby communicated a sense of sensory immersion. Olmsted wrote that the ideal park should be a place where people ‘may stroll for an hour, seeing, hearing and feeling nothing of the bustle and jar of the streets’. It stood in sharp contrast to its surroundings. He continued: ‘We want, especially, the greatest possible contrast with the streets and the shops and the rooms of the town… We want depth of wood enough about it not only for comfort in hot weather, but to completely shut out the city from our landscapes’. That was not to say that Central Park was at odds with the urban world entirely. Despite its escapist inclinations, the Greensward Plan was all about making the city liveable, creating a site where the toiling worker could spend quality time in restful repose. In fundamental terms, Olmsted saw the park as a somatic tonic for a built landscape in which residents suffered ‘functional derangements’ and most street trees had been ‘deformed by butcherly amputations’. It acted as a sanitising device for the city at large (especially its air) and also provided what he labelled as a ‘harmonising and refining’ palliative for the minds and bodies of human inhabitants.^20^

A health rationale also underscored park implementation schemes in Montreal, where the population mushroomed from 57,000 in 1851 to 300,000 in 1880 and 600,000 by WW1. Canada’s premier metropolitan centre and locus of industrial and financial activity, the city grappled with the deleterious consequences of rapid development, especially problems around waste and sanitation, fire risk and high-density housing. In calling for programmes of social and environmental reform, voices from various quarters articulated a sense of the city as a living organism, an urban metabolism beset by public health challenges and one in need of restorative greening. Writing in *The City Below the Hill* (1897), a sociological survey of an urban body in crisis, Herbert Ames talked of the ‘interdependence of society’ and the need to look at improving slum neighbourhoods, hygiene and urban degeneration as necessary priorities for the health and wealth of modern Montreal. As well as the illustrations playing out in city streets, inspiration also came from American example, London and Paris, and innovations elsewhere in Canada (especially the west). Underscoring the argument were the familiar axioms of breathing spaces and democratic access, put succinctly in a report from the City Engineer of Winnipeg: ‘The policy in all civilised countries [is] to reserve large areas of land where the citizens of all classes can escape from the noise and smoke of the crowded streets for pure air and recreation [but, in Canada] …no provisions were being made for lungs for the cities’.^21^

As with the other cities discussed here, the park idea in Montreal found fertile soil with municipal authorities looking to enhance civic infrastructure along sanitary lines and involved the remodelling of older areas (e.g. Place d’Armes Square [1840]) and the creation of brand new public reserves. An example of the latter, Mount Royal Park, was bought by the city in 1874 as a response to public lobbying over residential development and the denuding of timber resources for firewood. This greened piece of high ground in the centre of the city was used by locals for informal recreation and affectionately known as ‘the mountain’. A signal of the importance assigned to this project, civic leaders looked to Frederick Law Olmsted to design the park as a naturalistic hub around which city residents and the patients of the newly relocated *L’Hotel Dieu* hospital (1861) could take natural resort. Pastoralism reigned supreme at Mount Royal, though the site specifics of crafting a worker’s playground from the Laurentian shield saw Olmsted modify his plans on the basis of what he called ‘refined and delicate taste’ and ‘novel conditions’. This design, one of the last to be completed in a distinguished career of landscape uplift, saw Olmsted extrapolate on the preventative and curative benefits of park-making, not only for fresh air and exercise but, as he told Montreal’s civic leaders, ‘the power of scenery to eliminate conditions which tend to nervous depression or irritability’. Trees planted at the base of the hill created useful shade and amplified the sense of a park rising from city streets, while a winding carriage road took visitors to the lookout point in harmonious seclusion. Laying out his vision of ‘charming natural scenery’, Olmsted positioned Mount Royal as an unparalleled spatial prescription offering a ‘remedial way to enable men to better resist the harmful influences of ordinary town life’. It was, he argued, in a ‘medical phrase, a prophylactic and therapeutic agent of vital value’.^22^

Frederick Todd, New Hampshire-born landscape architect who worked in the Olmsted office in Massachusetts, played an important role in realising the Mount Royal project. Despatched to Montreal as the firm’s ‘plants man’, he oversaw the realisation of horticultural uplift and played an important role in reclaiming Beaver Lake, a marshy area seen as an unsanitary wasteland which was remodelled as a visitor attraction. Todd settled in the city and became a key figure in its evolving cityscape as an advocate of planting for health: becoming the city’s first registered landscape architect and one of the founders of the Town Planning Institute of Canada. Writing in ‘Character in Park Design’ (1905), he described the important role played by green space in serving the ‘mental, physical and moral’ needs of a progressive city, its naturalistic airs presenting a reflexive corrective to ‘all that is sordid and artificial’ in offering spatial refreshment to the ‘tired souls of city dwellers’.^23^

Two of Todd’s other commissions in Montreal strike as particularly relevant to the story of parks and its configuration of somatic urbanism. The first, was St Helen’s Island Park (1939) consciously established to cater for the needs of 100,000 working class urbanites in one of the most deprived districts who could reach it within walking distance of home. Envisaged as ‘the front door of the city’ due to its location on the St Lawrence and carried out as a public works project that used unemployed labourers, St Helen’s illuminated the multiple functions of the park as a demonstrator of civic virtue, device of horticultural uplift and repository of what he called ‘social dividends’. Using a familiar language of health enrichment, he pointed to its value as a restful space for residents grappling with the ‘bustle and strife of a large city’, for mothers and children ailing in ‘narrow tenements’, and for all citizens needing to breathe ‘more pure air than on the street’. Second, was the expansive remodelling of the Mount Royal district (1912), a garden suburb where an ordered blueprint of efficient circulation was overlaid on vacant plots immediately below the park that bore its name. Drawn up for the Canadian Northern Railroad, the ‘model city’ was conjured up as part of financing plans for a railroad tunnel under ‘the mountain’ via the sale of high-end residential lots, but also draw attention from civic leaders for its potential to re-imagine city space in utopian ways. Todd envisaged an idyllic conurbation with a wooded mountain backdrop, with a central railway station and ‘village green’ radiating from which were transit arteries of ordered, geometric precision. At the edges of the town, undulating roads marked out residential zones (here Todd saw curves not as a ‘waste of energy’ but a way of creating attractive neighbourhoods), while encircling the central district with 13 parks, described as ‘small squares and breathing places’, each designed to serve the needs of citizen health in this modern greened and airy city’.^24^

## Controlling the Citizen Body: ‘Tranquilising Medication’ and the Social Engineering of Green Space

Grand designs in somatic urbanism saw the park become assimilated into the modern city as a ubiquitous feature, to a vital breathing spaces for the democratic body to escape from factory time and the alienating ‘street canyons’ of industrial capitalism. Writing in the mid-1900s, Robert Moses, New York Parks Commissioner, hailed them as the ‘outward visible symbols of democracy’. That said, there were substantial caveats to the claims of green liberty laid out in park ideologies. Offering only a partial realisation of the communitarian virtues intrinsic to their civic function, things looked a little different on the ground. For a start, the sanitary terraforming of the city contained a series of problematic assumptions about what constituted progress, health and prosperity in urban industrialism. Accordingly, as Martin Gaskell explains, the establishment of new parks seemed more like ‘palliatives’ which provided neighbourhoods with some accessible open space yet failed to address the critical socio-economic needs of working-class city dwellers (especially in regards to housing). Hiding in plain sight was another problem: a top–down approach to urban greening that saw the solution to the paradox of modernity through the lens of social engineering and readily crossed the lines between prescription and proscription.^25^

Utopian visions of restorative scenery did not come from a blank canvas. Victoria Park was previously the resort of Chartist campaigners for parliamentary reform (who used Bonners Field as a rendezvous site) as well as criminal gangs, while Battersea Fields’ gypsy fairs, grazing commons and lively hostelries were frowned upon by civic authorities and wealthy developers looking to sanitise and homogenise city space. In New York, Irish, German and African American residents of a district that Olmsted condemned as ‘part of the straggling suburbs…filthy, squalid and disgusting’ were evicted. As Dorceta Taylor notes, the removal of marginal populations to make way for greensward gentrification was unsurprising as parks-in-the-making were typically located in neglected areas which were, most importantly, cheap to purchase. Moreover, city prerogatives for public health improvement also betrayed a strong element of class pacification at play. The gains were twofold. Land clearance enabled municipal authorities to erase, disempower or assimilate pariah communities, rebellious elements and informal economies. It also, reformers and medical professionals argued, made way for a remade space that would physically and psychologically refresh the workforce. In both cases, the economic and political health of the city was seen as being advanced. Viewed in these terms, the language of socio-environmental uplift implicit to somatic urbanism contained a series of assumptions about authority, power and social control. *What* was seen as healthy activity in the park was not a simple question. Neither was the issue of *who* decided such things. Nor, indeed, on behalf of *whom*.^26^

As an engineered landscape designed to promote public order and social cohesion, the park offered a carefully choreographed version of green liberty. Prominent in the minds of municipal planners and social reformers (who were predominantly middle class) was an intention to direct the leisure activities of the working classes. A trip to the park promised (in the minds of metropolitan authorities) a vast improvement to popular and ‘low and debasing’ amusements such as music halls, public houses and boxing matches. There was, however, more at stake than entertainment preferences. For the Select Committee on Public Walks, park creation represented a prophylactic against civic agitation: without adequate open spaces for ordinary folk to let off steam in, they argued, ‘great mischief must arise’. Viewed in this context, the ‘green liberty’ of the park spoke less of an extension of rights to the city and more of an attempt to dispel the potentially rebellious energies of a working-class citizenry. Also worth noting is the fact that the wide streets and open greensward of the sanitary city enabled more effective control and surveillance. Explicitly created to make it easier for law enforcement to travel through city environs and harder for disquieted districts to erect barricades, Haussmann’s wide boulevards served as symbolic and practical markers of state authority. Equally, as H. L. Malchow notes, London’s sweeping parklands ‘were more easily policed than the warren-like back courts and dark alleys of the impacted slum’. As such, the scattering of green enclaves across an urban geography presented a fitting accompaniment to the various institutional architectures of reform—schools, prisons, hospitals, asylums—that emerged as bastions of health, order and normative behaviour in the modern city.^27^

As Schrank and Ekici point out, the reformist zeal for remaking the city in healthful form invited a particular concern about the body as a site of vitality and vulnerability. It thus mattered acutely *what* people did in green space. The idea of the wholesome emerged as a particularly important vector when it came to the socio-environmental functionality of the park. Accordingly, when reformers picked carefully across the landscape of emparkment, a clear hierarchy emerged as to who was allowed to wander freely across *rus in urbe*. Framed by the democratic mantle of green liberty, the park was there for the benefit of all, an egalitarian amenity to serve the civic whole by harnessing its organic energy. At the same time, however, there remained distinct rules as to which members of the citizen body could navigate park space unencumbered. Flash points emerged around the proper role of the parks as places of public assembly, especially when it came to large gatherings and radical political figures (‘verminous persons’ in the park-keeper vernacular), while somatic urbanism left little room for those who fell under civic designations of the unsavoury, undeserving or deviant. After dark, especially, unwelcome demographic circulations from the likes of vagrants, prostitutes and criminals invited criticism of what Pierre Boitard called the ‘mysterious recesses’ that seemed to attract ‘people with bad morals and inveterate rogues’. The homeless—many of whom used parks as living spaces in increasingly privatised cityscapes—came under fire as threats to the restorative park experience on the grounds of unsociability and hygiene.^28^

For those who *did* gain the figurative keys to park gates, meanwhile, rights of use were decidedly different to rights of access. Social engineering, aligned with medical theory on the benefits of certain kinds of exercise, choreographed the recreational experience so that it focussed around gentle movement and thought. On this point, notions of embodied experience, sensory engagement and relational interaction shed useful light on the operation of the park as a health geography in practice. Walking, in particular, emerged as the principal conduit for improving well-being for the working classes (the elite, it seemed, could gather the same sensory ‘hit’ by riding in carriages). To fully benefit from the health-landscape encounter, visitors were encouraged to take in the scenery, gain olfactory stimulation from flora and cultivate a quietness of mind from the spacious silence of tree-lined meadows. This was an exercise not of idle enjoyment but obligation, expressed forcefully in the words of G. J. Romanes, who explained: ‘recreation is, or ought to be, not a pastime entered upon for the sake of pleasure which it affords, but an act of duty undertaken for the sake of the subsequent power which it generates, and the subsequent profit which it ensures’. Olmsted, likewise, saw the function of greensward as actively constitutional: ‘The park is not simply a pleasure-ground, that is, a ground to which people may resort to obtain some sort of recreation, but a ground to which people may resort for recreation in certain ways and under certain circumstances *which will be conducive to their better health’*. As with many medicines, one was not meant to enjoy a dose of the park too much.^29^

This sense of the socio-economic capital invested in the relational experience coalesced with theories of landscape value, the aesthetic worth of the ‘most healthful’ encompassed by the natural or pastoral form, what Olmsted called the most ‘soothing and reposeful’ of scenic styles. In *Public Parks and the Enlargement of Towns,* he spoke of how parks needed ‘the beauty of the fields, the meadow, the prairie, of the green pastures and still waters’ in order to cultivate ‘tranquillity and rest to the mind’. The manner by which slices of greensward offered up health to its imbibers was explained in transcendental and ephemeral terms. To Olmsted, this was a ‘poetic’ mechanism of uplift that crept ‘gradually and silently’ and brought therapeutic benefit through a process best described as osmotic. Scenery, he noted, had ‘an effect on the human organism by an action of what it presents to view, which action, like that of music, is of a kind that goes back of thought, and cannot be fully given the form of words’. Todd labelled this ‘unconscious or indirect recreation’. The quality of the healthful encounter was thus contingent on a syncretic relationship between *how* green space looked and *what* people did in park space.^30^

As shepherds of landscape amenity in a hybrid ecology of natural and social design, civic organs and park officials keenly asserted their rights to decide what was best for the people on the basis of moral, medical and municipal authority. Accordingly, when locals near Victoria Park asked for fetes, galas and fireworks, the Parks Board muttered about depreciating house prices and rowdy activity and instead recommended that visitors take a fine stroll and admire the orderly carpet planted borders and 32,000 new trees on display. Official rules in Halifax People’s Park, England (1857) banned park users from playing football, dancing, playing games and swimming. Wardens in New York’s Central Park reprimanded anyone straying off the paths to walk on the hallowed grass, using foul language, engaging in brawls or trying to sell goods. As Olmsted noted of Mount Royal: ‘if thousands of people are to seek their recreation upon it unrestrainedly, each according to his own special tastes, it is likely to lose whatever of natural charm you first saw in it’. In order to achieve its maximum potential as a therapeutic landscape, the onus remained on the cultivation of a calm and slow experience, preserving a world of ‘tranquilising medication’ in which the wanderer gleaned the healing benefits of space by exposure to sunlight, deep breathing and moderate exercise. With much invested in the power of greensward to heal the city body, civic authorities saw themselves as critical brokers of somatic urbanism.^31^

## Breathing Spaces and Rights to the City: Circulations of Health and History in the Twenty-first Century Park

*‘Breathing space in a city is quite as essential to the mental, moral and physical health of its people as building space’*.—William A. Stiles (1888)

*‘Everybody has a right to air, light, water and greenery’*.—Jean Baptiste Fonssagrives (1869)

These lines, written by two horticultural commentators on either side of the Atlantic, ably communicated the importance of the park as an operational device of somatic urbanism. Conjured as multi-purpose spatial prescription, the salubrious air and restorative mobility offered by the park situated it as a powerful site of green lungs and green liberty. It was at once set apart from the city, and yet firmly installed as a vital organ in making the urban body function. A curious blend of escapism and social engineering, it occupied a critical place in the modern city where the paradox of modernity was addressed by circulations of matter, metaphor and movement. Meanwhile, as part of an energetic urban metabolism, the park was never static. In the years since its foundational plantings, it proved an adaptable and evolving entity: one shaped by successive municipal authorities, interest groups and urban citizens (along with the ground itself) to fit the changing contours of the city body.^32^

Turning to the twenty-first century, the park remains firmly rooted in the fabric of metropolitan life, so much so that the *International Review of Landscape Architecture* contends that an urban centre without one *cannot* be a modern city. Wandered by generations of urbanites, it has become part of the vernacular urban experience, a meeting ground for social exchange and cultural amenity. According to Cranz, even if people do not use the park, they *want* to know it is there. Notions of environmental and social well-being, meanwhile, continue to loom large in articulations of its nature and purpose, while demographic projections that 68% of humans will live in cities by 2050 suggests an ongoing role as a multi-purpose therapeutic and sanitary landscape. The miasma theory of disease may have been replaced with an epidemiological view of medicine that turned the attention of public health professionals to the ‘great indoors’ in the early twentieth century, but, as Thomas Fisher notes, the holistic attention of nineteenth-century park planners to ecological and social matters presents a particularly useful model for dealing with the complex challenges of our present. In a world of anthropogenic climate crisis, biodiversity loss, urban densification and digital overload, the preventative (sanitary) and curative (medical) functions of the park lend it vital somatic functions. Scientific and medical evidence endorses the value of green space in filtering hydrocarbons, serving as noise and heat sinks, enhancing climate resilience through flood control and carbon capture; and supplying millions of humans with physical and psychological benefits through exercise and biotic connectivity.^33^

Parks, it seems, are good for cities. They are, however, not, uncomplicated places. For one thing, seeing them as a panacea for the urban condition at large fails to address the structural problems of an urban body disordered by unsustainable rubrics of growth and prosperity. A ‘sustainable park’ of the sort envisaged by Galen Cranz—networked and responsive to the urban needs of a new millennium—needs to sit in a city space that is programmed along similar lines. Today’s parks also face a range of limiting conditions that compromise their functionality as democratic healing spaces. Top–down, normative valuations of ‘healthful’ activity continue to choreograph citizen amenity and to rehearse a problematic authority founded on prescription and proscription, especially in terms of the treatment of transgressive behaviour and the criminalisation of poverty. Equally serious issues remain about inequities of access in terms of the proximity of parks to socio-economically deprived neighbourhoods; how inclusive parks are for BAME communities; and the social stratification of leisure. Complicated political economies of management, moreover, mean that many have intractable health problems of their own courtesy of massive funding cuts, while ailing ecosystem services and degraded facilities; ever-present development pressures that threaten to amputate public space; and various illimitable socio-environmental contaminants—from particulate pollution to knife crime—conspire to create the park not as a place of bucolic refuge but of urban decay.^34^

These contrasting visions of rejuvenation and apoplexy were particularly illuminated by the coronavirus pandemic. In the time of COVID, the essential role played by green space in a built environment where many residents lack outdoor space at home was starkly elucidated. In London’s Victoria Park, this was written into the material landscape in the socially distanced ‘desire paths’ plotted across park lawns by locals taking their allotted walks and in conversations with user groups as to the sensory and health benefits of spending time *in* nature *and* during a global pandemic. Back in the mid-1800s, Octavia Hill had evocatively called the park an ‘outdoor sitting room’ and, 150 years on, facing the spectre not of cholera but of another airborne disease, citizens made important connections between the positive circulations of air, exercise, wellness and the right to roam. Other aspects of the COVID park encounter, meanwhile, highlighted health inequalities and social regulation at work in public space, not least in Victoria Park, where gates were initially locked to visitors (prompting a huge outcry) and the policing of social distancing along ageist and ethnic lines showed the gap between park-making for democratic health and a fully realised civic realm where, as David Harvey contends, citizens ‘hold the right to remake the city’.^35^

COVID offered a new twist on an old story of park-making, health and the city, one which shined the spotlight on the need to rework urban ecologies to create better ventilation, safe transit corridors and meaningful community access to green space. A textured understanding, meanwhile, of the circulations of health and history seems like another essential ‘building block’ for liveable cities of the future. A critical meeting place of matter and metaphor, (to return to Masterson-Agar’s allusion) the park is a ‘text’ whose temporal contours need to by teased out, interrogated and contextualised in order to properly ‘remake’ the city. Contemporary urban planning has much to say about the importance of ‘place-making’, a participatory process used to describe the design and management of public space that is community-led and fosters healthy connections between people and place. This, especially, invites an important contribution from scholars in medical and environmental history who can speak to the eco-cultural dialectics of urban space and to explain the tracks of association that connect plants, play and people in a lively (and sometimes challenging) conversation across time. As this article has illuminated, a walk in the park has *always* involved far more than an idle wander, taking in, instead, a complex and connected world of somatic urbanism and a critical health geography with a vital connection to city bodies past, present and future.^36^

## Footnotes

1 Gregg Mitman, ‘In Search of Health: Landscape and Disease in American Environmental History’, *Environmental History*, 2005, 10, 184.

2 Araceli Masterson-Algar, *Ecuadorians in Madrid: Migrants’ Place in Urban History* (New York: Palgrave, 2016), 83. Peter Clark, Marjaana Niemi and Catharina Nolin, eds, *Green Landscapes in the European City, 1750-2010* (London: Routledge, 2010), 1. Recent urban environmental histories of this type include: Catherine McNeur, *Taming Manhattan: Environmental Battles and the Antebellum City* (Cambridge: Harvard University Press, 2014); Kara Murphy Schlichting, *New York Recentered: Building the Metropolis From the Shore* (Chicago: University of Chicago Press, 2019); Bill Luckin and Peter Thorsheim, *A Mighty Capital Under Threat: The Environmental History of London* (Pittsburgh: University of Pittsburgh Press, 2020); Harold L. Platt, *Building the Urban Environment: Visions of the Organic City in the United States, Europe, and Latin America* (Philadelphia: Temple University Press, 2015). For a more medical frame of reference, see: Tom Koch, *Cartographies of Disease: Maps, Mapping and Medicine* (Redlands: ESRI Press, 2005); Sara Jenson, *The Topography of Wellness: How Health and Disease Shaped the American Landscape* (Charlottesville: University of Virginia Press, 2021).

3 Galen Crantz, *The Politics of Park Design: A History of Urban Parks in America* (Cambridge: 2020 [1982]), 3–59; Elizabeth Grosz, ‘Bodies-Cities’, in Heidi Nast and Steve Pile, eds, *Places Through the Body* (Basingstoke: Routledge, 1998), 242. Short investigations into parks as ‘lungs’ can be found in: Karen R. Jones, ‘“The Lungs of the City”: Green Space, Public Health and Bodily Metaphor in the Landscape of Urban Park History’, *Environment and History,* 2018, 24, 39–58; John L. Crompton, ‘Evolution of the “Parks As Lungs” Metaphor: Is It Still Relevant?’, *World Leisure Journal*, 2017, 59, 105–123. On gardens and spatial justice, see: C. Rogers, E. Straughton, A. Winchester and M. Pieraccini, eds, *Contesting Common Land: Environmental Governance Past and Present* (London: Earthscan, 2011); S. Martin Gaskell, ‘Gardens for the Working Class: Victorian Practical Pleasure’, *Victorian Studies,* 1980, 23, 479–501; Shelley Egoz, Karsten Jorgensen and Deni Ruggeri, eds, *Defining Landscape Democracy* (Cheltenham: Edward Elgar, 2018); Abigail Gilmore, ‘The Park and the Commons: Vernacular Spaces for Everyday Participation and Cultural Value’, *Cultural Trends,* 2017, 26, 34–46.

4 Richard Dennis, *Cities in Modernity: Representations and Productions of Metropolitan Space, 1840-1930* (Cambridge: Cambridge University Press, 2008), xiii; Anne Whiston Sprinn*, The Granite Garden: Urban Nature and Human Design* (New York: Basic Books, 1984), xii, 262; Catherine Ward Thompson, ‘American Parks and Contemporary Needs’, *Landscape Journal,* 1988, 17, 2, 4, 9; Peter Thorsheim, ‘Green Space and Class in Imperial London’, in Andrew Isenberg, ed, *The Nature of Cities: Culture, Landscape, and Urban Space* (Rochester: University of Rochester Press, 2006), 34. On modernity in comparative context, see: M. Levin, *et al.*, *Urban Modernity: Cultural Innovation and the Second Industrial Revolution* (Cambridge: MIT Press, 2010) and for an analysis of its problematics as a category, Keir Waddington, ‘Problems of Progress: Modernity and Writing the Social History of Medicine’, *Social History of Medicine,* 2021, 34, 1053–67. Histories of urban greening include: Hazel Conway, *People’s Parks: The Design and Development of Victorian Parks in Britain* (Cambridge: Cambridge University Press, 1991); R. Rosenzweig and E. Blackmar, *The Park and the People: A History of Central Park* (Ithaca: Cornell University, 1992); Dorothee Brantz and Sonia Dumpelmann, eds, *Greening the City: Urban Landscapes in the Twentieth Century* (Charlottesville: University of Virginia Press, 2019).

5 Martin Melosi, *The Sanitary City: Environmental Services in Urban America From Colonial Times to the Present* (Pittsburgh: University of Pittsburgh Press, 2008); Thompson, ‘American Parks’, 9. The phrase ‘somatic urbanism’ appears in a short blog on queer urban poetics which explores how a poet walks and writes about New York, but is only mentioned in the title and is not defined as a term (see: Davy Knittle, ‘James Schuyler’s Somatic Urbanism’ (9 January 2018). Available online at: https://jacket2.org/commentary/bodies-cities-part-1-queering-geographic-information).

6 W. M. Gesler, ‘Therapeutic Landscapes: Medical Issues in Light of the New Cultural Geography’, *Social Science and Medicine,* 1992, 34, 735–46; A. Williams, ed, *Therapeutic Landscapes: The Dynamic Between Place and Wellness* (Maryland: University Press of America, 1999); Sarah L. Bell, *et al.*, ‘From Therapeutic Landscapes to Healthy Spaces, Places and Practices: A Scoping Review’, *Social Science & Medicine,* 2018, 196, 123–130; A. C. Gattrell, ‘Therapeutic Mobilities: Walking and “Steps” to Wellbeing and Health’, *Health & Place,* 2013, 22, 98–106.

7 For medical histories of the body, see: J. Robb and O. Harris, *The Body in History* (Cambridge: Cambridge University Press, 2013); L. Kalof and W. Bynum, eds, *A Cultural History of the Human Body* (London: Bloomsbury, 2010); S. Sheard and H. Power, eds, *Body & History: Histories of Urban Public Health* (Aldershot: Ashgate, 2000). On outdoor space and medicine, see: Julie Collins, *The Architecture and Landscape of Health: A Historical Perspective on Therapeutic Places, 1790-1940* (London: Routledge, 2020); Clare Hickman, *Therapeutic Landscapes: A History of English Hospital Gardens Since 1800* (Manchester: Manchester University Press, 2013); Carla Yanni, *The Architecture of Madness* (Minneapolis: University of Minnesota Press, 2007).

8 Richard Sennett, *Flesh and Stone: The Body and the City in Western Civilization* (London: Faber & Faber, 1994), 255–270; Pamela Gilbert, *Mapping the Victorian Social Body* (New York: SUNY), 6; Dennis, *Cities in Modernity,* 2; Sabine Barles, ‘The Urban Metabolism of Paris and Its Region’, *Journal of Industrial Ecology,* 2009, 13, 898–913.

9 Neil Arnott, *Elements of Physics, Natural Philosophy: General and Medical* (London: Longman & Roberts, 1833), 452; Victor Hugo, *Les Miserables* (New York: New American Library, 1987), 1259–61; Charles Dickens, *Our Mutual Friend* (London: Chapman & Hall, 1865), II/1; Henry Roberts, *Home Reform: Or What the Working Classes May Do to Improve Their Dwellings* (London: The Society for Improving the Condition of the Labouring Classes, 1852), 5; Considerant quoted in Patrice De Moncan, *Le Paris D’Haussmann* (Paris: Les Éditions du Mécène, 2012), 10; P. Joyce, *The Rule of Freedom*: *Liberalism and the Modern City* (New York: Verso, 2003), 65.

10 Vanessa Taylor talks about circulation and hydrology in ‘Water and Its Meanings in London, 1800-1914’, in *A Mighty Capital Under Threat*, 155–76, while Christopher Hamblin explores discourses around ‘managing matter’ and oxygenation flows in ‘The City as a Chemical System? The Chemist as Urban Environmental Professional in France and Britain, 1780-1880’, *Journal of Urban History,* 2007, 33, 702–28. J. C. Loudon, ‘Hints on Breathing Places for the Metropolis, and for Country Towns and Villages, on Fixed Principles’, *Gardener’s Magazine*, 1829, V, 686–90; Thomas Wakley, ed, *The Lancet*, 2 vols (London: George Churchill, 1829), 869; Comte de Rambuteau, *Memories* (Paris: Colman Levy, 1905), 377; Jean-Charles Adolphe Alphand, *Les Promenades de Paris* (Paris: J. Rothschild, 1867–1873), 59; Clare Hickman, ‘“To Brighten the Aspect of Our City Streets and Increase the Health and Enjoyment of Our City,”: The National Health Society and Urban Green Space in Late 19^th^ Century London’, *Landscape and Urban Planning,* 2013, 118, 112–9; Edwin Chadwick, Report on the *Sanitary Conditions of the Labouring Population of Great Britain* (Edinburgh: Edinburgh University Press, 1965 [1842]), n.p.

11 Cobbett’s Parliamentary Debates During the Second Session of the Fourth Parliament of the United Kingdom of Britain and Ireland, 1808, XI, 1124–5; J. C. Loudon, ‘Letter to the Editor’, *Literary Journal*, 1803, 2, 739; Robert Angus Smith, *A Centenary of Science in Manchester* (London: Taylor & Francis, 1883), 62.

12 For a discussion of civic republicanism in the philosophy of Frederick Olmsted from a political science perspective, see Scott Roulier, ‘Frederick Law Olmsted: Democracy by Design, *New England Journal of Political Science*, 2009, 4, 311–43.

13 Octavia Hill, ‘Space for the People’, *Macmillans,* 1825, 32, 329; J. F. Murray, ‘The Lungs of London’, *Blackwoods,* 1839, 46, 212–27.

14 Report from the Select Committee on Public Walks, Parliamentary Papers 1833, xv.448 vols, 10, 6; *Parliamentary Papers, House of Commands and Command, Vol. 16, Reports From Commissioners, Session 5 February-27 August 1839*, 80.

15 Dennis, *Cities in Modernity,* 1; Baron Ernouf, *L’Art des Jardins* (Paris: Rothschild, 1868), 352.

16 Ernest Guinier, *Promenades, Parcs Jardins Paysagers* (Annecy: Abry, 1904), 13–4, 202.

17 New York *Mirror*, 22 July 23 1842; *New York Daily Times,* 4 June 1853.

18 F. L. Olmsted, ‘Justifying Values of a Public Park’ in Charles E. Beveridge and Carolyn F. Hoffman, eds, *The Papers of Frederick Law Olmsted, Supplementary Series, Vol. 1: Writings on Public Parks, Parkways and Park Systems* (Baltimore: Johns Hopkins University Press, 1997), 336; Sprinn, *Granite Garden,* 34; *Scientific American,* 19 November 1859, 37; Caroline Kirkland, *Holidays Abroad, or Europe from the West* (New York: Baker & Scribner, 1849), 93–94; Olmsted, *Walks and Talks,* 133–41, 78–83.

19 F. L. Olmsted, ‘Notes on the Plan of Franklin Park and Related Matters’, in *Papers of FLO*, (1886), 467; Frederick Law Olmsted, ‘Public Parks and the Enlargement of Towns’, *Papers of FLO*, 179, 188, 182–3; Thomas Fisher, ‘Frederick Law Olmsted and the Campaign for Public Health’, *Places* (November 2010). Available online at: https://placesjournal.org/article/frederick-law-olmsted-and-the-campaign-for-public-health/?cn-reloaded=1.

20 Olmsted, ‘Public Parks’, 192–3, 189, 183, 198.

21 Herbert Ames, *The City Below the Hill: A Sociological Study of a Portion of the City of Montreal, Canada* (Montreal: Bishop Engraving and Printing Co, 1897), 4; ‘Winnipeg Works: Annual Report of Mayor Ruttan, City Engineer’, *Manitoba Free Press*, 1892.

22 Frederick Law Olmsted to Mount Royal Park Commissioners, 12 November 1875, Frederick Law Olmsted Papers, Library of Congress, Washington, DC; Frederick Law Olmsted, Mount Royal, Montreal (New York: Putnam’s 1881), 22, 13.

23 Frederick G. Todd, ‘Character in Park Design’, *Canadian Municipal Journal,* 1905, 10, 321–2.

24 ‘Historic Saint Helen’s Island Park’, *Municipal Review of Canada*, 1938, 34. 14, 20; Todd, ‘Character’, 321; ‘The Miracle of Modern Colonization in Canada’, *Dominion*, 148, quoted in Nancy Pollock-Ellwand, ‘The Prolific Interpreter of the Olmsted Vision: Frederick G. Todd, Canada’s First Landscape Architect’, *Planning Perspectives,* 2019, 34, 191–214.

25 Robert Moses, ‘The Moses Recipe for Better Parks’, *Recreation,* 1956, 49, 215; Sprinn, *Granite Garden*, 57; Gaskell, ‘Gardens’, 491.

26 Second Annual Report. Description of the Central Park. Board of Commissioners of the Department of Public Parks. New York. Year ending 1 May (1859), 59–68; Dorceta Taylor, ‘Central Park as a Model for Social Control: Urban Parks, Social Class and Leisure Behavior in Nineteenth-Century America’, *Journal of Leisure Research,* 1999, 31, 440.

27 Select Committee on Public Walks, 8; H.L. Malchow, ‘Public Gardens and Social Action in Late Victorian London’, *Victorian Studie*s, 1985, 29, 99.

28 Schrank and Ekici, *Healing Spaces,* 1–2; Pierre Boitard, *Manuel d’Architecture des Jardins* (Paris: Roret, 1854).

29 Romanes quoted in Peter Bailey, *Leisure and Class in Victorian England* (London: Routledge & Kegan Paul, 1978), 67; Frederick Law Olmsted, General Order for the Organization and Routine of Duty of the Keepers’ Service of the Central Park. Document No. 43. Minutes. New York, Department of Public Parks, 31 March 1873.

30 Olmsted, ‘Justifying Values of a Public Park’; 344–5; Olmsted, ‘Public Parks’, 23; Olmsted, ‘Notes on the plan of Franklin’, 522; Todd, ‘Character in Park Design’, 321–2.

31 Olmsted, ‘Mount Royal’, 26.

32 William Stiles, ‘The Attack on the City Hall Park’, *Garden & Forest*, 1888, 135; Jean Baptiste Fonssagrives, *L’Education Physique des Jeunes Filles* (Paris: Hachette, 1869); Cranz, *Politics*, 223.

33 Topos, ed, *Parks: Green Spaces in European Cities* (Basel: Birkhäuser, 2002), 5; Cranz, *Politics,* 223.

34 Cranz, *Politics*, xiii–ix. See also: Isabelle Anguelovski and James Connelly, eds, *The Green City and Social Injustice* (London: Routledge, 2021).

35 Octavia Hill, ‘Open Spaces’, *The Commons, Open Spaces & Footpaths Society 1865-1903* (London: COPFS, 1965), 113; David Harvey, ‘The Right to the City’, *International Journal of Urban and Regional Research,* 2003, 27, 939–941.

36 P. J. Ellery, J. Ellery and M. Borkowsky, ‘Toward a Theoretical Understanding of Placemaking’, *International Journal of Community Well-Being,* 2021, 4, 55–76.

